# Nanoporous fluorescent sensor based on upconversion nanoparticles for the detection of dichloromethane with high sensitivity†

**DOI:** 10.1039/d0ra08058f

**Published:** 2020-12-24

**Authors:** Haiyan Wang, Shiping Zhan, Xiaofeng Wu, Lingqiong Wu, Yunxin Liu

**Affiliations:** Department of Information and Electrical Engineering, Hunan University of Science and Technology Xiangtan 411201 China; Department of Physics and Electronic Science, Hunan University of Science and Technology Xiangtan 411201 China; School of Computer and Information Engineering, Hunan University of Technology and Business Changsha 410205 China lyunxin@163.com xfwuvip@126.com

## Abstract

A sensor with high sensitivity and response rate is still lacking in the detection of poisonous and volatile chemicals. Here, we report a highly sensitive nanoporous fluorescence sensor based on core@shell upconversion nanoparticles (UCNPs) for the detection of dichloromethane. UCNPs were deposited on porous anodic alumina oxide (AAO) templates supported by glass slides to form a thin film-like gas sensor in which UCNPs with active shells exhibit intense background-free fluorescence and simultaneously high optical sensitivity, while an AAO template acts as a porous substrate for UCNPs to increase the absorption capacity for molecules to be tested. A detection limit of 2.91 ppm was obtained for dichloromethane based on this sensor at room temperature. The involved response mechanism was attributed to lowered surface fluorescence quenching and scattering of UCNPs by dichloromethane.

## Abstract 2

We report a highly sensitive nanoporous fluorescence sensor based on core/shell upconversion nanoparticles (UCNPs) for the detection of dichloromethane. UCNPs are deposited on porous anodic alumina oxide templates to form a thin film-like gas sensor.

## Graphical abstract

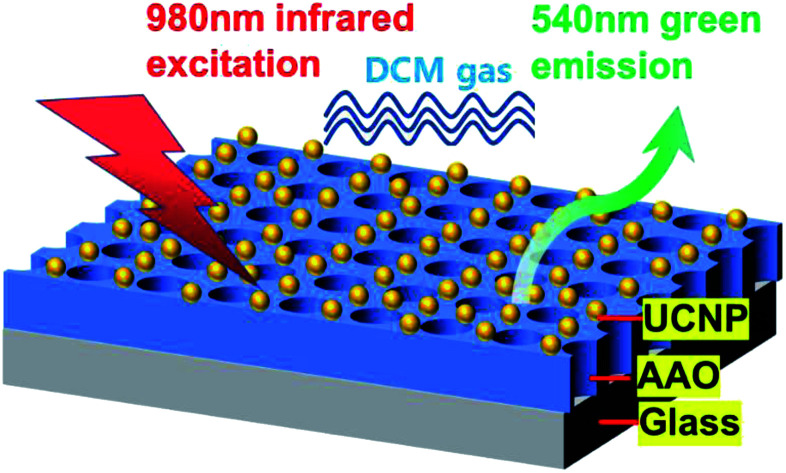

## 1. Introduction

As one of the most powerful luminescent nanocrystals, upconversion nanoparticles (UCNPs) have received extensive attention owing to their advantages of multicolor, narrow bandwidth, and no photobleaching,^1^ due to which they have been extensively applied for background-free imaging, biological detection labeling,^2,3^ temperature sensing,^4^ and solar cells with enhanced infrared absorption.^5^ They have been confirmed to be very efficient in the detection of glutathione,^6^ DNA,^7^ disease biomarkers,^8^ metal ions,^9–11^ and dyes.^12^ Recently, this specific material is reported to be sensitive to NH_3_, CO, NO, and H_2_S.^13–16^ However, traditional UCNPs suffer tremendous fluorescence quenching problem, which seriously limits their further application in the highly sensitive detection of volatile organic compounds.

Volatile organic compounds (VOC) such as dichloromethane, which are widely used in the pharmaceutical industry and in the production of cine-films, are harmful to the environment and cause human liver and nervous system injury or even cause cancer.^17–20^ As per the safety standards for occupational exposure to dichloromethane (DCM), the concentration of DCM at TWA-75 ppm and a ceiling of 500 ppm was recommended using related functional departments.^21^ Therefore, the precise detection of DCM is very desirable and important in the related industries. Currently, DCM gas sensors based on semiconductors,^22–25^ carbon nanotubes, and polymers^26,27^ have been reported. Lee *et al.* reported a SnO_2_-based gas sensor for DCM measurement that possessed high sensitivity (ppm level) but suffered from ultrahigh operation temperature (350 °C).^22,23^ Winadda *et al.* proposed a DCM sensor based on carbon nanotubes that can be easily operated at room temperature but has a high detection limit.^28^ In recent years, considerable advances have been achieved in optical or photoelectric DCM sensors due to the superiority of optical signals in fast response, low loss, and flexible optical spectra modulation. Han *et al.* have introduced a DCM detector based on near-infrared absorptive sensing and obtained high resolution,^29^ while the optical sensor based on fluorescence quenching behavior in Cd metal–organic framework is found to be highly efficient.^30^ However, some problems, such as photobleaching under the excitation of ultraviolet light and the anti-interference problem, should be resolved for these optical sensors to exhibit high performance.^29,30^ Lopes *et al.* reported a bioluminescent (lux) bacterial bioreporter for the detection of DCM and achieved a detection limit of 1.0 ppm under exposure to vapor phase and 0.1 ppm under exposure to liquid phase with response times of 2.3 h and 1.3 h, respectively.^31^

Here, we report a nanoporous fluorescent sensor based on UCNPs with high sensitivity and low detection limit along with the advantages of low energy consumption, stable photochemical properties, and sensing at room temperature. The sensor based on NaGdF_4_:Yb,Er@NaYF_4_:Yb active-core@active-shell UCNPs (ACAS-AAO) exhibit higher sensitivity and response rate for the detection of trace amount of DCM than the one based on UCNPs with inert shells. This is due to the following facts. First, with the increasing concentration of DCM, active-core@active-shell UCNPs shows higher intensity than core UCNPs with/without inert shells.^32^ Second, ACAS-AAO shows an exponential dependence of the fluorescent intensity on the concentration of DCM, while UCNPs with/without inert shells show a linear dependence of the fluorescent intensity on the concentration of DCM. In addition, AAO templates can improve the local molecule exchange of DCM through capillarity action such that the supported UCNPs can rapidly and efficiently capture DCM molecules. Based on this ACAS-AAO sensor, a detection limit of 2.91 ppm can be achieved in the detection of DCM. The involved response mechanism was ascribed to reduced surface fluorescence quenching and scattering of upconversion nanocrystals by DCM.

## 2. Experimental

### 2.1. Chemical and reagents

Rare earth oxides (Gd_2_O_3_, Yb_2_O_3_, Er_2_O_3_, Y_2_O_3_), oleic acid (OA), octadecene (ODE, 90%), NaOH (98%), NH_4_F (98%), cyclohexane, hydrochloric acid, methanol, acetic acid, and dichloromethane, were purchased from afar (Tianjin) chemical effective company and used without further purification. AAO templates were purchased from Shenzhen Topology Film Technology.

### 2.2. Synthesis of UCNPs

UCNPs with/without shells were synthesized by the solvothermal method in the solvent system of OA/ODE with slight modification, which is described in detail in the ESI.†^33^

### 2.3. Gas sensing measurement

The gas sensing performance of the nanoporous fluorescent sensor was investigated at room temperature (30 °C, 303 K) with a relative humidity of 40%. The above sensor was transferred into a glass container sealed with a rubber stopper. Then, DCM was introduced into the container by a syringe needle, while the flux was controlled by a flow counter. The whole device was placed in a spectrometer for real-time indication of the concentration of DCM gas. The response (%) was defined as (*I*_D_ − *I*_0_)/*I*_0_, in which *I*_0_ is the upconversion emission intensity without dichloromethane, and *I*_D_ is the upconversion emission intensity with dichloromethane.

Note that UCNPs synthesized by the solvothermal method in OA/ODE system still had a thin coating of OA after washing with ethanol and distilled water. The nanoporous fluorescent sensor based on OA-coated UCNPs was hydrophobic, and its fluorescent intensity was dependent on the concentration of DCM but independent of the relative humidity in the range of 0%–70%. Furthermore, the sensor based on active core@active shell UCNPs has good selectivity and reversibility, with the data being presented in the ESI (Fig. S6 and S7).†

### 2.4. The characterization of UCNPs and AAO

The size and shape of the lanthanide-doped core and core@shell UCNPs were determined by a transmission electron microscope (JEM-2100F operating at the voltage of 200 kV). The structure of the anodized aluminum template with and without UCNPs was characterized by a scanning electron microscope (Field emission JSM-6700F). The composite structure AAO@core@shell can be excited by 980 nm infrared laser and, upon excitation, it emits a green light at 540 nm. The fluorescence spectra were obtained using the Hitachi-F2700 spectrometer.

## 3. Result and discussion

### 3.1. The morphology, structure, and fluorescence spectra of core and core@shell UCNPs

**Fig. 1:**
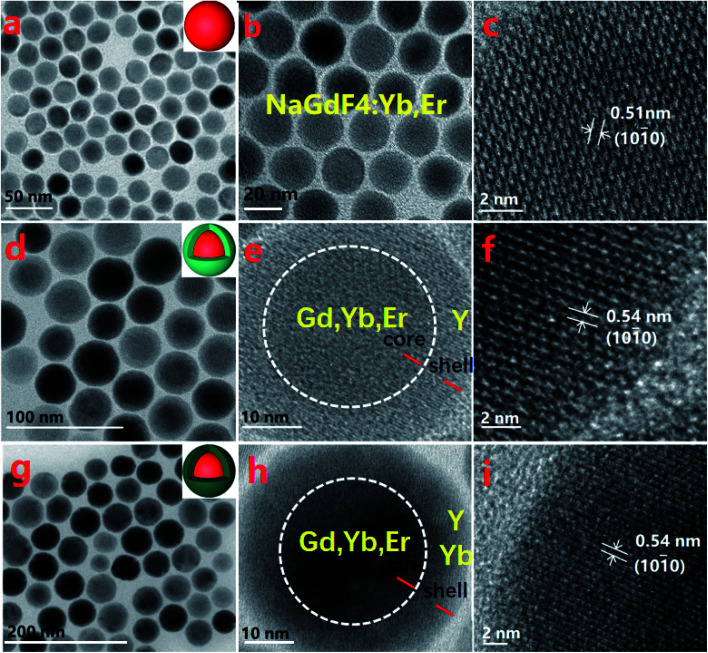
TEM and HR-TEM images of core and core@shell UCNPs. (a–c) NaGdF_4_:Yb,Er core UCNPs; (d–f) NaGdF_4_:Yb,Er@NaYF_4_ core@shell UCNPs; (g–i) NaGdF_4_:Yb,Er@NaYF_4_:Yb core@shell UCNPs.

**Fig. 2:**
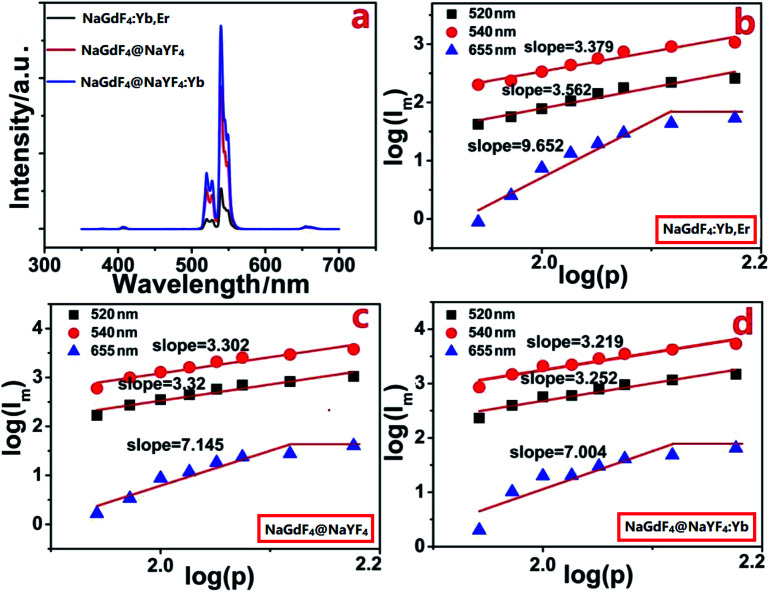
(a) The fluorescence spectra of core and core@shell UCNPs. Dependence of fluorescence intensity (*I*_m_) on the excitation power density (*P*) for (b) NaGdF_4_:Yb,Er core UCNPs, (c) NaGdF_4_:Yb,Er@NaYF_4_ core@shell UCNPs, and (d) NaGdF_4_:Yb,Er@NaYF_4_:Yb core@shell UCNPs.

The morphology and particle size of NaGdF_4_:18%Yb^3+^/2%Er^3+^ nanoparticles with and without shells were determined from TEM images shown in Fig. 1. From Fig. 1a–c, it is clear that NaGdF_4_:18%Yb^3+^/2%Er^3+^ nanoparticles possess spherical shape, hexagonal phase (Fig. S1†), a uniform particle size of 25 nm, and exposed NaGdF_4_ crystal planes (111) with an interplanar spacing of 0.51 nm. After epitaxial growth with a NaYF_4_ inert shell of 7.6 ± 0.5 nm (Fig. 1d–f), the spherical shapes and uniform particle size were retained; however, NaYF_4_ crystal planes (111) were exposed with an interplanar spacing of 0.54 nm. NaGdF_4_:Yb,Er@NaYF_4_:Yb ACAS nanoparticles, shown in Fig. 1g–i, exhibit spherical shapes and a shell thickness of 8.9 ± 1.0 nm, but NaYF_4_:5%Yb crystal planes (111) were exposed with an interplanar spacing of 0.54 nm. All these core-only and core@shell UCNPs can be dispersed in cyclohexane.^34^ These UCNPs can emit strong green light centered at 540 nm under the excitation of 980 nm near-infrared light (Fig. 2a). Clearly, the core@shell nanoparticles exhibit a much higher fluorescent intensity than bare core nanoparticles,^35^ while the nanoparticles with active shells possess the highest fluorescent intensity. In addition, these nanoparticles emit red light centered at 655 nm and violet light centered at 410 nm of much lower intensities. This indicates that the emitter Er^3+^ ion had a dominant 4f_*n*_-electronic population on the level of ^4^S_3/2_ through the non-radiation relaxation of ^4^F_7/2_ → ^4^S_3/2_ that further goes down to the ground state for emitting green light.^36^ The photoluminescence mechanisms of these nanoparticles can be deeply revealed by studying the dependency of the luminescent intensity on the excitation power. According to Auzel's theory, the luminescent intensity (*I*) is proportional to excitation power (*P*): *I* ≈ *P*^*n*^, where *n* is the number of infrared excitation photons for producing a visible emission photon. The dependence of luminescent intensity on the excitation power is shown in Fig. 2b–d for NaGdF_4_:Yb,Er, NaGdF_4_:Yb,Er@NaYF_4_, and NaGdF_4_:Yb,Er@NaYF_4_:Yb, respectively, under a weak excitation power density from 0.30 to 0.42 W mm^−2^ with an interval of 0.02 W mm^−2^.

It can be determined from the slope value of Fig. 2b–d that absorption of ∼3 infrared photons of 980 nm was required to produce a green photon of 540 nm, which is different from the commonly reported conditions, in which two infrared photons are absorbed by UCNPs to generate a green photon of 540 nm. This indicates that the energy exchange among electrons was almost negligible in photon upconversion processes under weak excitation. Thus, the slope value indicates a weak energy exchange among electrons of the emitter Er^3+^ ions that is beyond the relationship between the emission intensity and excitation power described by Auzel's equation. In this condition, we could actually observe the simplest upconversion process of a single Er^3+^ ion with negligible energy transfer between Er^3+^ ions. Furthermore, it is observed from Fig. 2b–d that the slope value of both green (540 nm and 520 nm) and red (655 nm) emissions are inversely proportional to the photoluminescence efficiency.

NaGdF_4_:Yb,Er@NaYF_4_:Yb active core@active shell nanoparticles show the highest photoluminescence efficiency (Fig. 2a)^32^ but exhibit the lowest slope value (Fig. 2d). In other words, NaGdF_4_:Yb,Er@NaYF_4_:Yb active core@active shell UCNPs had an obviously higher upconversion efficiency for single Er^3+^ ion than NaGdF_4_:Yb,Er and NaGdF_4_:Yb,Er@NaYF_4_UCNPs. Hence, it is noteworthy that upconversion nanoprobes usually exhibit high sensitivity when excited by a relatively low power density because the molecules to be tested breaks down the independent upconversion process of a single emitter. However, the strong energy exchange between emitters is commonly generated by excitation with high power density because for breaking the energy exchange between emitters usually requires additional energy or stronger external impact.

As a result, it is important to reveal the upconversion characteristics of upconversion nanoprobes under a low power density to determine their potential sensitivity as fluorescent probes. Undoubtedly, from Fig. 2, it is clear that NaGdF_4_:Yb,Er@NaYF_4_:Yb active core@active shell nanoparticles should possess the highest sensitivity as optical probes under excitation with low power density. However, the practical effect will be presented in the detection of DCM (Fig. 4).

### 3.2. The nanoporous structure of DCM gas sensor

**Fig. 3:**
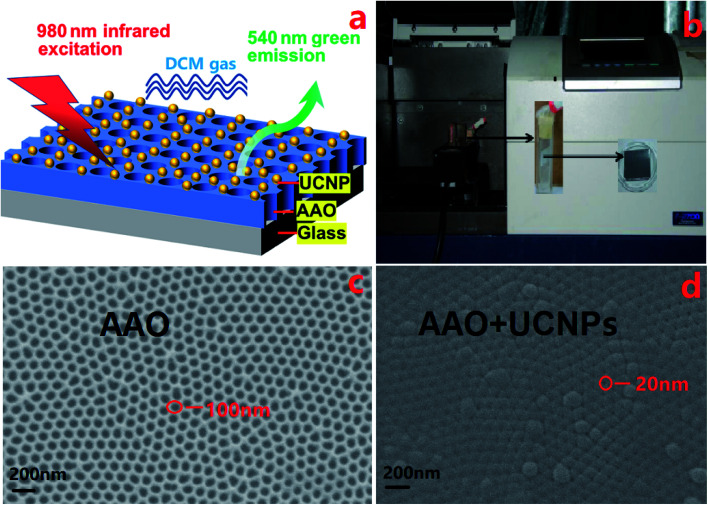
(a) A model diagram of (ACAS-AAO) nanoporous sensor. (b) The real sensor ACAS-AAO is placed in a sealed container with DCM gas which is located in a spectrometer (Hitachi F-2700). SEM images of AAO templates without UCNPs (c) and with UCNPs (d).

To detect DCM, a sensor was designed and fabricated based on UCNPs. UCNPs were first deposited on AAO templates supported by glass slides to form a thin film-like gas sensor (Fig. 3a). Subsequently, the film sensor was placed in a glass container sealed with a rubber stopper. Then, DCM gas was introduced into the container by a syringe needle while controlling the flux by a flow counter. The whole device was placed in a spectrometer for real-time indication of the concentration of DCM gas (Fig. 3b). In addition, the structure of AAO templates with/without UCNPs is presented in Fig. 3c and d, respectively. The figure reveals that the porous AAO template had an average pore diameter of 100 nm and an average wall thickness of 20 nm, while the AAO template with UCNPs had an average pore diameter of 19 nm and an average wall thickness of 101 nm. This indicates that UCNPs were successfully coated on AAO templates to generate a porous film with the same pore sites as in the AAO template but with much smaller pore size.

### 3.3. Sensing behaviors of DCM gas sensor based on UCNPs and AAO template

**Fig. 4:**
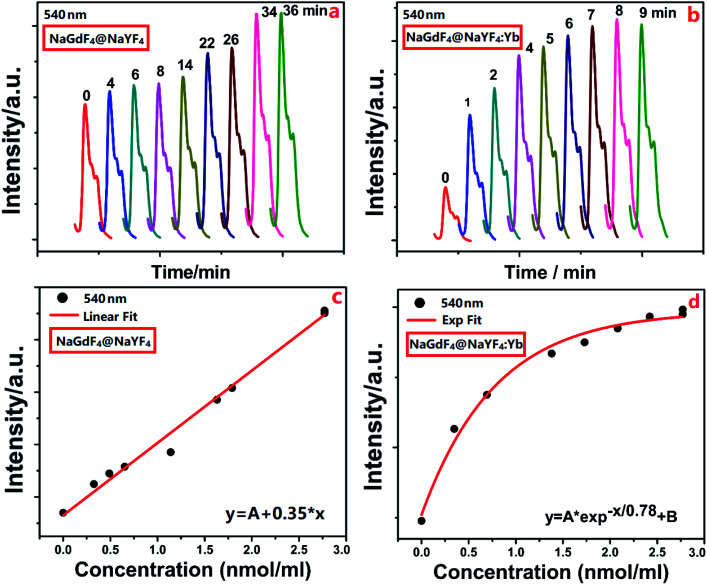
The detection of DCM gas based on porous AAO supported UCNPs sensor. Dependence of fluorescence intensity (540 nm) on the introduction time of DCM gas into the sealed container based on (a) the sensor with NaGdF_4_:Yb,Er@NaYF_4_ UCNPs and (b) the sensor with NaGdF_4_:Yb,Er@NaYF_4_:Yb UCNPs. Dependence of upconversion emission intensity (540 nm) on the concentration of DCM gas based on (c) the sensor with NaGdF_4_:Yb,Er@NaYF_4_ UCNPs and (d) the sensor with NaGdF_4_:Yb,Er@ NaYF_4_:Yb UCNPs.

Based on the above sensor, the concentration of DCM gas can be determined through real-time monitoring of the green fluorescence spectra (540 nm) under the excitation of infrared light of 980 nm. Fig. 4a and b show that the emission intensity of UCNPs increases on extending the import time of DCM gas. This enhancement was ascribed to the lowered surface scattering for NaGdF_4_:Yb,Er@NaYF_4_ NPs based sensor (Fig. 4a) and lowered surface quenching and scattering for NaGdF_4_:Yb,Er@NaYF_4_:Yb NPs based sensor (Fig. 4b). Thus, the active shell with the sensitizer Yb^3+^ ions had a strong influence on the luminescence of the core. However, the active shell contacts closely with the DCM molecules. Consequently, the active shell actually plays a role of an energy bridge between the fluorescent probe (emission core) and the DCM molecules to be tested such that the NaGdF_4_:Yb,Er@NaYF_4_:Yb NPs-based sensor exhibits a much higher response rate than the NaGdF_4_:Yb,Er@NaYF_4_ NPs-based sensor. The dependence of emission intensity on the concentration of DCM gas is shown in Fig. 4c and d. A linear relationship with a coefficient of 0.35 was observed for NaGdF_4_:Yb,Er@NaYF_4_-based sensor, while an exponential relationship with a coefficient of 0.78 was observed for NaGdF_4_:Yb,Er@NaYF_4_:Yb.

**Fig. 5:**
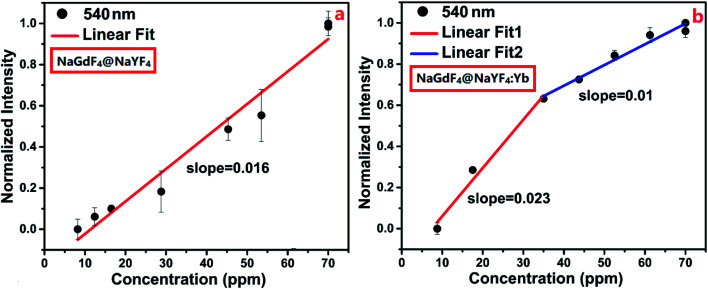
The linear fitting curve of the emission intensity *vs.* the concentration of DCM gas based on the nanoporous sensors.

The linear dependence of normalized emission intensities on the concentration of DCM gas is shown in Fig. 5. It is clear that a linear relationship with a coefficient of 0.016 was observed for a NaGdF_4_:Yb,Er@NaYF_4_-based sensor, and a detection limit of 4.91 ppm was determined. The linear relationship with a coefficient of 0.023 and 0.01 was observed for a NaGdF_4_:Yb,Er@NaYF_4_:Yb-based sensor at the low concentration and a relatively higher concentration of dichloromethane, respectively, while a detection limit of 2.91 ppm was determined. The dependence of fluorescence intensity (540 nm) on the concentration of DCM gas was measured and is shown, for the glass slide supported UCNPs sensor (without AAO template), in Fig. S3 and S4 of the ESI.† It is quite clear that AAO templates play the role of efficient absorbers for stably capturing DCM molecules around UCNPs.^37^ For the condition without AAO templates (Fig. S3 and S4 in the ESI†), DCM molecules flow through UCNPs randomly such that UCNPs cannot sense the linear increase of the concentration of DCM gas. As a result, a non-monotonous dependence of emission intensity on the concentration of DCM gas was observed (Fig. S4 in the ESI†).

**Fig. 6:**
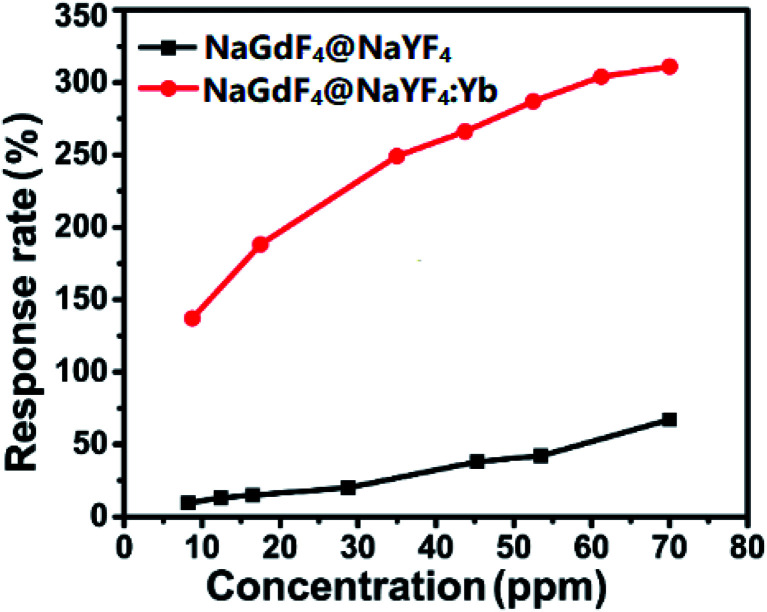
The response rate of nanoporous sensors to the varying concentration of dichloromethane gas.

The response curves of the sensor based on UCNPs with/without active shells are presented in Fig. 6. From the figure, it is quite clear that a NaGdF_4_:Yb,Er@NaYF_4_:Yb-based sensor presents a much higher response to the concentration variation of DCM gas, while the sensor based on UCNPs with active shells shows a maximum response of 308%, which was 4.5 times greater than the sensor based on UCNPs with inert shells. In comparison with previously reported sensors for detecting DCM molecules (Table 1), these nanoporous sensors based on the AAO template supported upconversion nanocrystals exhibit a relatively lower detection limit to DCM at room temperature.

**Table 1:** The comparison of different gas sensor for DCM^a^

DCM detection sensor					
Sensing material	*T* (°C)	Rage (ppm)	Limit (ppm)	Signal type	Reference
SnO_2_	350	0.5–30	0.1	Resistance	25
		0.1–0.8	0.1		22 and 23
TiO_2_	250	10–50	None	Resistance	24
Polymer (PECH)	20	2000–18 000	294	Frequency	26
Polymer	25	None	125	Frequency	27
Carbon nanotube	RT	1700–2150	None	Resistance	28
Methylobacterium	20	1–100	1	Bioluminescent	31
	280	0.1–100	0.1		
**UCNPs C/S (Y, Yb)**	**RT**	**2.91–70**	**2.91**	**Spectrum**	**This work**
**C/S (Y)**	**RT**	**4.91–70**	**4.91**		

a RT: room temperature; None: no report in the article.

## 4. Conclusion

A highly sensitive nanoporous fluorescent sensor based on UCNPs was demonstrated for the detection of DCM. UCNPs were deposited on AAO templates supported by glass slides to form a thin film-like gas sensor in which UCNPs with active shells exhibited intense background-free fluorescence and simultaneously high optical sensitivity, while anodic alumina oxide template supplies a porous substrate for UCNPs to increase the absorption capacity for molecules to be tested. Based on this sensor, a detection limit of 2.91 ppm was obtained for DCM, and the involved response mechanism was ascribed to the lowered su 1rface fluorescence quenching and scattering of upconversion nanocrystals by DCM.

## Conflicts of interest

There are no conflicts to declare.

## Supplementary Material

RA-011-D0RA08058F-s001
