# Supplementary material for: Nanoporous fluorescent sensor based on upconversion nanoparticles for the detection of dichloromethane with high sensitivity
Source: RSC Adv. 2020 Dec 24;11(1):565–71. doi: 10.1039/d0ra08058f (PMC8691116; doi:10.1039/d0ra08058f)
Supplement: RA-011-D0RA08058F-s001 [file RA-011-D0RA08058F-s001.pdf]

Electronic Supplementary Material (ESI) for RSC Advances.
This journal is © The Royal Society of Chemistry 2020

Nanoporous fluorescent sensor based on upconversion nanoparticles for the detection of dichloromethane with high sensitivity

Haiyan Wang, [a,1] Shiping Zhan, [b,1] Xiaofeng Wu, [a,c,*] LingqiongWu, [b] Yunxin Liu [c,*]

[a] Department of Information and Electrical Engineering and [b] Department of Physics and Electronic Science, Hunan University of Science and Technology, Xiangtan 411201 China. [c] School of Computer and Information Engineering, Hunan University of Technology and Business, Changsha 410205, China.

1.Experimental section:

1.1 Chemical and reagents

Rare earth oxides Gd_2O_3, Yb_2O_3,Er_2O_3, Y_2O_3, oleic acid (OA),octadecene (ODE,90%, purchased from afar (tianjin) chemical effective company, NaOH(98%),NH_4F(98%) cyclohexane,，hydrochloric acid, dichloromethane, deionized water, methanol, acetic acid. The reagents used in this study are all analytical pure.

1.2 Synthesis of UCNPs

UCNPs were synthesized by solvothermal method. First, the rare earth chloride was dissolved with excessive dilute hydrochloric acid and put on the magnetic stirrer to stir and evaporate four-fifths of the solvent, and the rare earth chloride ($RECL_3$,RE:Gd,Yb,Er,Y) was obtained.$GdCl_3$ (0.195mmol), $YbCl_3$ (0.05mmol), $ErCl_3$ (0.005mmol) and 2ml OA were added to A flask for 40 minutes at 145 degrees. Then 2ml ODE was added and remained for 20 minutes at the same temperature until solution clarified in pale yellow was formed. The temperature was adjusted to 60 degrees for thermal insulation. Add 2ml OA and 2ml octadecene (ODE) in B flask and stir for one minute, then add 5ml NaOH and NH_4F methanol mixture at 100 degrees for 40min to remove the methanol in the solution. The particles in the solution are small and uniform, and adjust the temperature to 60 degrees for thermal insulation. In the C flask, 8ml OA and 8ml ODE were added, and the stirring interval was heated to 325 degrees, and then the temperature was kept for 15 minutes. Nitrogen was injected into the reaction process to protect it. Solutions A and B were successively dropped into solution C, and then reacted at 325 degrees for 60min. When they were cooled to room temperature, they were put into the centrifuge for centrifugation. After centrifugation, cyclohexane was used for preservation. All these core-only and core@shell UCNPs can be well dispersed in cyclohexane, which can also be dispersed in ethanol and water after surface

modification with polyethylene glycol (PEG). These UCNPs are synthesized by previously reported method except the slight tuning of the doping ion.

2. Supplementary figures

2.1 The XRD of core and core-shell UCNPs

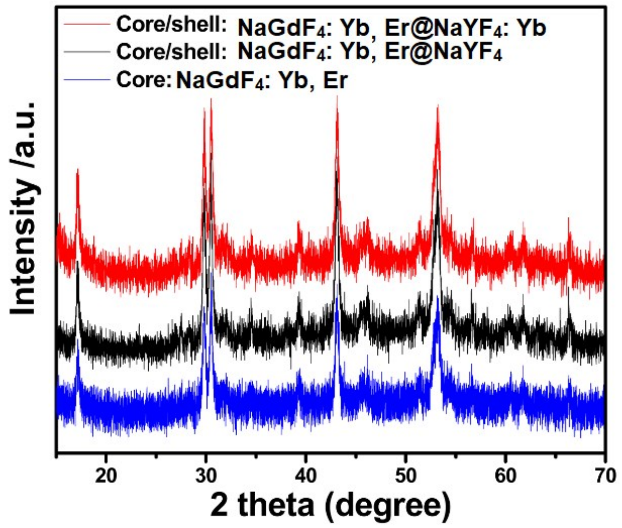

Figure. S1. XRD pattern of $NaGdF_4$:Yb,Er core UCNPs, $NaGdF_4$:Yb,Er@$NaYF_4$ core@shell UCNPs and $NaGdF_4$:Yb,Er@$NaYF_4$:Yb core@shell UCNPs. From the XRD pattern, we can see the core and core-shell UCNPs belong hexagonal phase.

2.2 The structure of sensing system.

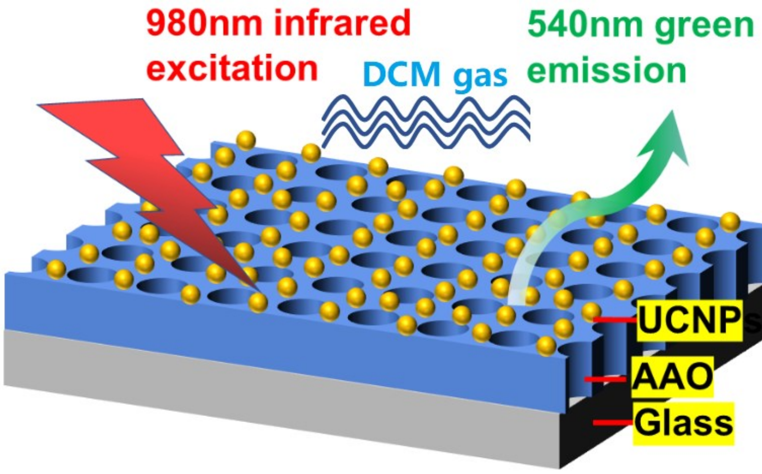

Figure. S2. A model diagram of (ACAS-AAO) nanoporous sensor.

In order to detect DCM, a sensor is designed and fabricated based on UCNPs. UCNPs are first deposited on AAO templates supported by glass slides to form a thin film-like gas sensor Subsequently, the film sensor is placed in a glass container sealed with a rubber stopper. DCM gas is introduced into the container by a syringe needle while the flux is controlled by a flow counter. The whole device is placed in a spectrometer for real-time indicating of the concentration of DCM

gas.

2.3. Sensing behaviors of DCM gas sensor based on glass supported UCNPs

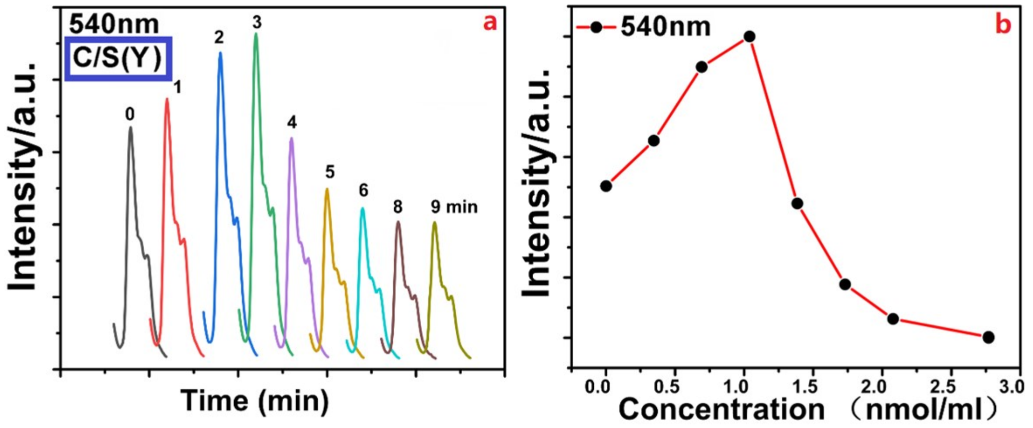

Figure. S3. The detection of dichloromethane gas based on glass slide supported UCNPs sensor (without AAO templates). (a)Dependence of fluorescence intensity (540nm) on the introduction time of dichloromethane gas into the sealed container based on the sensor with$NaGdF_4$: Yb, Er@$NaYF_4$ UCNPs and (b)Dependence of upconversion emission intensity (540nm) on the concentration of dichloromethane gas based on the sensor with $NaGdF_4$: Yb, Er@$NaYF_4$ UCNPs.

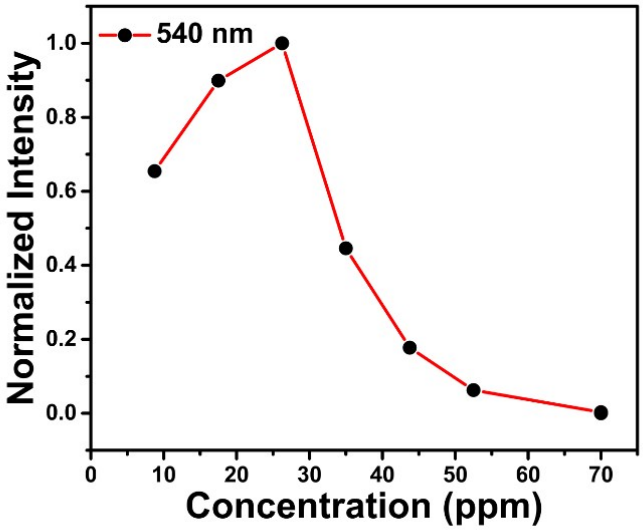

Figure. S4. The curve of the normalized emission intensity vs. the concentration of DCM based on the glass supported UCNPs sensors.

2.4 The selectivity of DCM gas sensor based on AAO supported active-core @active-shell UCNPs.

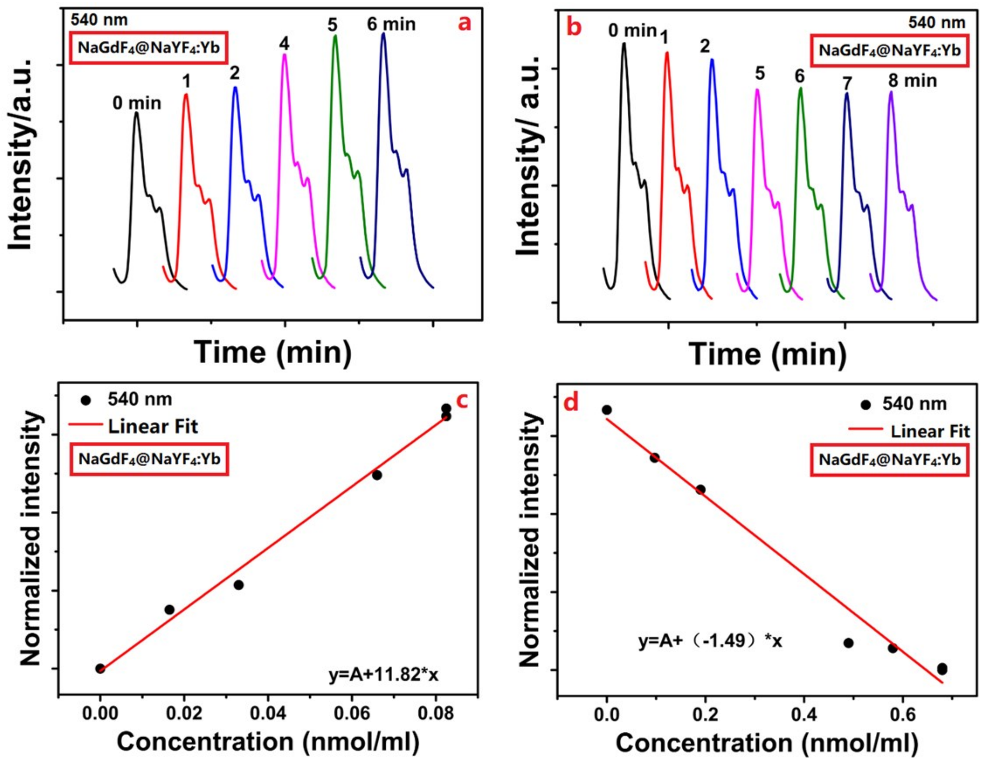

Fig. S5. The detection of DCM gas based on porous AAO supported active-core@active-shell UCNPs. Dependence of fluorescence intensity (540nm) on the introduction time of (a) acetic acid gas and (b) methanol gas into the sealed container based on the sensor with $NaGdF_4$:Yb,Er@$NaYF_4$:Yb UCNPs, respectively. Dependence of upconversion emission intensity (540nm) on the concentration of (c) acetic acid gas and (d) methanol gas based on the sensor with $NaGdF_4$:Yb,Er@ $NaYF_4$:Yb UCNPs, respectively.

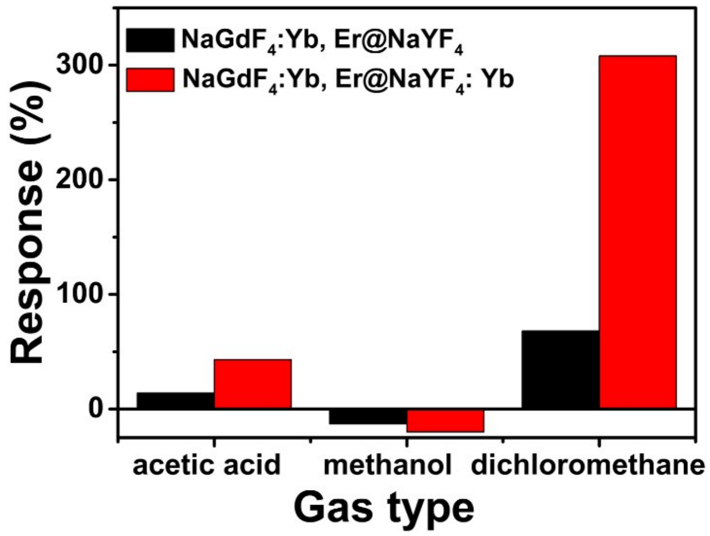

Figure. S6. The response of the sensor based on $NaGdF_4$:Yb,Er@$NaYF_4$ UCNPs and the sensor based on $NaGdF_4$:Yb,Er@$NaYF_4$:Yb UCNPs to acetic acid gas, methanol gas, and DCM gas, respectively.

From the Figure S5, we can see the sensor based on core@shell UCNPs had the highest response

value to dichloromethane compared with methanol, acetic acid, the result confirm that the sensor shows good selectivity toward dichloromethane.

2.5 The reversibility of DCM gas sensor based on AAO supported $NaGdF_4$: Yb, Er@$NaYF_4$: Yb core shell UCNPs.

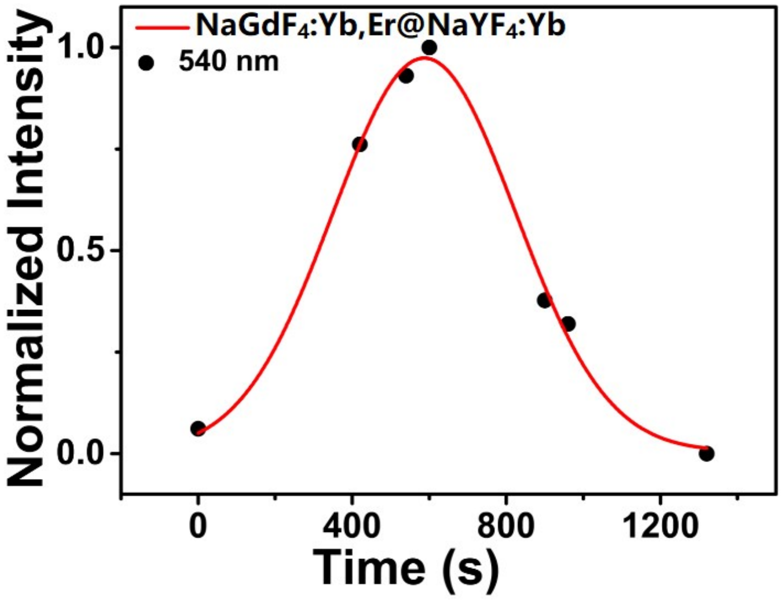

Figure. S7. The reversibility of dichloromethane gas sensor based on the AAO template supported $NaGdF_4$:Yb,Er@$NaYF_4$:Yb UCNPs. The rising edge presents the increasing process of the DCM gas while the falling edge shows the decreasing process of the DCM gas.
